# The shaping of adolescent physical activity habitus: The role of family sports culture

**DOI:** 10.1371/journal.pone.0336905

**Published:** 2025-12-17

**Authors:** Haoyuan Zheng, Zubing Xiang, Wenli Yang, Haojun Xi

**Affiliations:** School of Physical Education, Chongqing University, Chongqing, China; ISSEP Kef: Universite de Jendouba Institut Superieur du Sport et de l'Education Physique du Kef, TUNISIA

## Abstract

Based on Bourdieu’s habitus theory, this study revealed the connotation, function, and shaping mechanism of physical activity habitus. Utilizing a stratified random cluster sampling survey conducted across 16 public undergraduate universities in Chongqing, China, the data were quantitatively analyzed using factor analysis and OLS regression analysis models to explore the impact of family sports culture on the formation of adolescent physical activity habitus in primary school, middle school, and high school. The study constructed two main dimensions of adolescent physical activity habitus: self-report physical behavioral capacity and physical behavioral inclination. Further research indicates that family sports culture may maintain a certain correlation with the two dimensions of adolescents’ physical activity habits throughout their entire educational trajectory. Moreover, it is noteworthy that factors such as family economic status, paternal education level, gender, and urban-rural disparities appear to demonstrate weaker associations with adolescents’ physical activity habits when compared to the influence of family sports culture. In conclusion, this study suggests that family sports culture during the primary school stage may represent a potentially critical factor in shaping adolescents’ physical activity habits. Therefore, it is imperative for all sectors of society to pay more attention to the construction of family sports culture.

## Introduction

### The impact of physical activity on the physical and mental health

A study showed that 81% of 1.6 million adolescents aged 11–17 from 146 countries did not engage in physical activity in 2016. The lack of physical activity among adolescents has become a global issue [1].

Physical activity participation is related to physical and mental health in children and adolescents. Children who maintain sedentary behavior and lack physical activity tend to have higher composite risk factor scores for cardiovascular disease compared with children who engage in regular physical activity [2–4]. Researchers in the field of psychology have found physical activity effectively mitigates psychological distress while enhancing resilience and well-being [5–8]. Students who continuously engaged in school sports throughout middle school demonstrated better mental health outcomes compared to those who never participated in school sports [9]. Sedentary behavior is a risk factor for increasing anxiety symptoms in adolescents, while breaking up prolonged bouts of sitting could reduce this risk [10].

### The sociological influence mechanism of adolescents’ participation in physical activities

From the sociological view, scholars have explored the sociological mechanism underlying adolescents’ physical activity behavior from diverse perspectives. Within the growth process of adolescents, family cultures are the source of crucial predispositions to participate which have lasting effects [11]. Parents and other family members have an important impact on promoting adolescent physical activity [12,13]. Bourdieu, a renowned French sociologist, posited that individual behaviors were socially and historically determined, being interconnected rather than isolated [14]. Bourdieu believed that habitus constituted the system of behavioral inclination continuously, shaped by actors in their actual behaviors in daily life, representing an invisible set of rules [15]. Previous studies have found that physical activity started as a confluence between the physically active habitus [16]. Children’s individual habitus, as bearing the experiences of their familial backgrounds, provided them with certain desires to participate in physical activity [17].

### Habitus and physical activity habitus

Habitus is what individuals bring to and is/are shaped within fields. It is the product of an individual’s social experiences in the world, a product of time and history [15], which becomes inscribed in our bodies [18]. Habitus in the field of physical activity comes from individuals or groups engaging in physical activities, internalized into people’s consciousness through time and practice, which mobilizes and directs their behavior, eventually externalizing into a behavioral pattern. The concept of the habitus is an intermediate step between the structure and the agent, which establishes mediations to go beyond explanatory reductionism that only considers one of these aspects [19], with a large number of individual case studies. According to Bourdieu’s conceptualization of “cultural capital, fields, and habitus” [18], the efforts required to incorporate cultural capital into a specific field are influenced by the initial provision of this capital, which is “inherited” by children through early socialization in the family core. Research indicated that adolescent habitus, as a carrier of family background experiences, provided certain desires for their participation in physical activity, which were influenced by the family environment [17]. Stuij used habitus theory to study the socialization of sports and exercise, and found an interesting phenomenon: in the lower social class, the habitus is influenced by the extended family, the physical education teacher and peers, resulting in a broad range of less strictly ordered activities, undertaken at different places [20]. There is limited empirical research on adolescent physical activity behavior through the lens of habitus theory. Specifically, methods for measuring physical habitus lack depth. Bourdieu considered habitus as a social structure, a structured material, a framework containing internal forms, integrating the dynamic world or specific fields, thereby shaping an individual’s perception and actions in this world [14]. Habitus is the embodied history based on past experiences (i.e., the trajectory of class status evolution), particularly influenced by early experiences, including family experiences such as gender roles, household goods, consumption patterns, and parent-child relationships [15]. The physical activity habitus is‘simultaneously physiological (endurance, aerobic capacity), cognitive (motor skills, coordination, game sense), social (teamwork, following rules), and emotional based on the concept of habitus (pushing oneself, managing pain)’ [16]. Based on previous research, we can conclude that physical activity habitus is an unconscious system of physical behavioral inclination, which should include selective tendencies for physical activity and the strength of practice ability. Self-reported physical activity capacity and physical behavioral inclination may be the main external manifestations of physical activity habitus. Therefore, we can indirectly assess the intensity of physical activity habitus and then validate them through empirical research.

### Family sports culture and adolescent physical activity habitus

Family sports culture is recognized as a significant factor in shaping adolescent physical habitus [21] and inclination towards physical activity activities [17]. Current scholars have explored the significant role of family or parents in shaping adolescents’ physical activity habitus from multiple perspectives. Social support, a crucial concept in the field of social sciences, encompasses tangible and instrumental support, resource and informational support, as well as emotional and psychological support [22]. Social support is predominantly measured using self-report scales. The Multidimensional Scale of Perceived Social Support (MSPSS) is currently one of the most widely used measurement tools in the field of social support research [23]. The scale comprises three dimensions: family support, friend support, and significant other support. As a critical component of family support, parental social support (PSS) refers to the conscious efforts made by parents to influence their children’s behaviors and activity participation through involvement, encouragement, discussion, and the provision of activity-related opportunities. Its multidimensional nature is reflected in the dynamic process of parent-child interaction [24]. Parental social support contributes to the development of autonomous motivation and decision-making capabilities in children regarding physical activity, thereby promoting more proactive and dynamic engagement in leisure-time exercise among children and adolescents [25]. Researchers studying adolescent physical activity behavior from the perspective of habitus theory have noted the relationship between family sports culture and adolescents’ physical activity behavior. Bourdieu categorized cultural capital into three forms: embodied cultural capital, objectified cultural capital and institutionalized cultural capital. Scholars have categorized the familial factors influencing Chinese adolescents’ sports participation from various theoretical perspectives, and subsequent empirical studies have confirmed that family sports cultural capital exerts a significant impact on their engagement in physical activities [26]. Social support is typically conceptualized as an external resource accessible to individuals, while “habitus” refers to an internalized, embodied, and durable system of dispositions that shapes an individual’s perceptions and modes of action. It emphasizes a dynamic system of behavioral tendencies capable of explaining the generation of practical strategies across different social fields, thereby contributing to a deeper understanding of the generative mechanisms underlying adolescents’ physical activity behaviors.

The resources available in a child’s family (among his/her parents) may influence the child’s daily agency, both in a direct situational way through the resources available for the child’s use ‘here and now’, but also in a more indirect socialization-based way where earlier family capital influenced practice has given the child certain personal resources and dispositions, i.e., capital and habitus, for interaction and agency in the present. Study indicated that family functioning played a vital role in helping to shape children’s dispositions towards physical activity [27]. Babkes’ study suggested that children’s perceptions of what their parents do and their attitudes toward those actions are crucial for their psychosocial outcomes. This reflects the potential psychosocial causal paths of family cultural capital in PA [28]. If parents do not engage in physical activity, adolescents are four times more likely to be inactive [29]. At the same time, social class is closely related to adolescents’ habitus, with children from higher socioeconomic backgrounds being more likely to engage in regular sports and exercise activities [21]. Indicators of parental socioeconomic status, such as family economic welfare and parents’ education level, are also associated with lower levels of physical inactivity and reduced participation in sports among adolescents [30]. Parents with higher socioeconomic status tend to be more actively involved in their children’s participation in sports and physical exercise [31]. These factors work together to influence adolescents’ physical activity habitus, and the methods and strategies parents use to encourage their children’s participation in physical activity are often influenced by their own social experiences [32].

Parents influence the sports-related lifestyles of their children in a number of ways [33], and specifically by (1) being role models [34]; (2) introducing their children to sport [35]; (3) encouraging them to take part in sport; (4) providing transport and equipment [36], and (5) by their mere interest in sport, manifested in joint activities such as hiking tours and by making sport a topic of conversation in the family [37].

### Present study

The focus of this study is to measure adolescents’ physical activity habitus through assessments of self-report physical behavioral capacity and physical behavioral inclination, and to explore its relationship with family sports culture. Studies have shown that adolescents’ participation in physical activity has social historicity. The habitus theory holds that such group disparities in adolescents’ physical activity behavior are mainly manifested in different behavioral capacities and behavioral inclinations. Physical activity habitus are a kind of invisible rules (a stable behavioral tendency system formed during the process of social history) that are continuously formed by the action subjects in sports social practice, and they have class differences and social historicity. They mainly include two dimensions: Physical behavioral capacity and Physical behavioral inclination. Physical behavioral capacity refers to an individual’s level of physical exercise engagement, including the comprehension of rules, application of techniques and tactics, and management of basic sports injuries; Physical behavioral inclination denotes an individual’s willingness to engage in physical exercise, including initiative in active participation and disposition to create conditions for physical activity. Through the research on cultural capital theory, we have discovered the fact that families exert a profound influence on adolescents’ physical activity behavior, and it cannot be ignored. Bourdieu’s cultural capital and habitus theory explain the underlying mechanisms responsible for the group differences observed in family sports culture and adolescents’ physical activity behavior. Existing research has indicated that physical activity habitus may encompass an individual’s predisposition to engage in physical activity, their motor competencies, and their understanding of its underlying rules. Although previous researches have widely shown that physical activity behavior possessed by parents and family cultural capital has an important impact on adolescents’ physical activity behavior, there remains a dearth of comprehensive discussion regarding the influence of families on adolescents’ physical habitus [38]. Besides, the measurement for assessing physical habitus was not confirmed either. Therefore, based on previous research and literature, this study aims to empirically analyze the influence mechanism of family sports culture on the shaping of adolescents’ physical habitus.

## Method

### Study design

Aligned with our research objectives, this study employed a retrospective non-experimental design. It investigates the influence of family sports culture factors on adolescents’ physical activity habits across different educational stages by surveying Chinese university students’ recollections of their primary, middle, and high school experiences, combined with their current physical activity practices.

#### Ethics approval and consent to participate.

The Ethics Committee of the School of Physical Education at Chongqing University approved this study on July 20, 2022. The approval number is 19YJC890048. This research was conducted in strict compliance with the relevant laws and ethical standards of China.The participants provided their written informed consent to participate in this study. Names of respondents were not included in the study to ensure anonymity. All methods were carried out in accordance with declaration of Helsinki.

### Study setting

This research was initiated in July 2022 with theoretical analysis and preliminary preparations. Over a three-month period, the survey questionnaire was developed through multiple rounds of expert review. Subsequently, electronic surveys were distributed to 16 public universities in Chongqing Municipality, China during November-December 2022.

### Study population

A total of 3,280 first- and second-year university students participated in this survey (freshmen = 1,644; sophomores = 1,636). In China, students at this academic level are typically aged 18–20 years. A total of 3,280 questionnaires were collected. After eliminating invalid responses (incomplete or logically inconsistent answers), 3,090 valid questionnaires were retained, comprising 41.42% male (n = 1,280) and 58.58% female (n = 1,810) participants. This retrospective longitudinal survey required participants to recall experiences from three educational stages: primary school (ages 6–12), middle school (ages 12–15), and high school (ages 15–18). To enhance response validity, we obtained a confidentiality certificate from the School of Physical Education at Chongqing University, ensuring data privacy protection. The informed consent procedure explicitly stated that no one except the research team would have access to individual responses.

### Sample size determination and sampling techniques

Stratified random cluster sampling method was employed to survey first- and second-year undergraduate students from 16 public universities in Chongqing. The total sample size was determined using the empirical rule requiring the number of valid questionnaires to be at least 10 times the number of items in the scale. The sample allocation for each university was proportional to the total enrollment numbers (approximately 167,000 students combined) during the 2021 and 2022 academic years. To account for potential questionnaire refusals or invalid responses, we expanded the sample size by approximately 10% to ensure the adequacy of ultimately valid responses.

### Data collection instruments and procedures

Data were collected through a structured questionnaire comprising three sections: the first covered basic socio-demographic characteristics, the second assessed the situation of adolescent physical activity habitus shaping, and the third captured retrospective accounts of family sports culture experiences during primary, middle, and high school periods. The electronic questionnaire was distributed by physical education teachers during class sessions and collected immediately upon completion. All data were entered into an electronic database and verified through dual-entry procedures to ensure accuracy.

### Reliability

To ensure research reliability, this study utilized a forced-choice 4-point Likert scale with the following response options: (a) Strongly Agree; (b) Agree; (c) Disagree; (d) Strongly Disagree for both the adolescent physical activity habitus scale (see Table 2) and the family sports culture scale (see Table 6), instead of the traditional 5-point scale containing a neutral option. This approach was adopted for specific social science considerations: while respondents often find it easier to express “strongly agree” or “strongly disagree” positions, accurately distinguishing between “slightly agree” and a neutral stance can be challenging. The tendency to default to a middle “neutral” option may lead to central tendency bias, potentially reducing the scale’s discriminative validity and statistical power. The 4-point scale simplifies cognitive processing, reduces respondent burden, improves survey efficiency and data quality, while minimizing central tendency bias.

The Cronbach’s alpha coefficients for the two dimensions of the adolescent physical activity habitus scale - “self-reported physical activity capacity” and “physical behavioral inclination” – were 0.885 and 0.916, respectively. For the family sports culture scales across primary, middle, and high school stages, the coefficients were 0.909, 0.934, and 0.932, respectively, indicating high reliability of the questionnaire measurements. The following steps were employed to secure the instrument’s validity. However, it must be acknowledged that the lack of reliable data from repeated tests is a limitation of this study.

### Validity

During the questionnaire design process, we carefully considered the characteristics of different respondent groups to ensure that all designed questions would enable accurate selection based on actual circumstances for all populations, including those who typically do not engage in physical activities. For individuals who are not regularly physically active, their inclination and capacity for physical activity are generally weak. For instance, when responding to the question “If you don’t engage in physical activity for a week, you feel something is missing in your life”, since they ordinarily do not participate in physical activities, the absence thereof would not significantly impact their lives, making them more likely to select “Disagree” as their answer. By adopting this approach, we aimed to maintain questionnaire conciseness and clarity while ensuring all respondents could provide answers based on a clear understanding of the questions, thereby enhancing survey validity.

The physical activity habitus measurement scale and family sports culture measure developed in this study adhered to processes including literature review, expert consultations, and preliminary research. An expert committee related to sport participatory behaviors was established to assess the content validity of the survey instruments, processing four rounds of expert checks. The questionnaire was revised and improved based on feedback from experts. The results of the final round of expert validity assessment are presented in Table 1. All invited experts held academic titles of associate professor or higher in relevant fields and are recognized as influential scholars within their disciplines. The questionnaire was originally developed by our research team and has not been previously published elsewhere.

**Table 1:** Statistical results of expert validity assessment.

Evaluation Item	Highly Appropriate	Basically Appropriate	Neutral	Slightly Inappropriate	Inappropriate
Overall Questionnaire Design	2 (14%)	12 (86%)	0	0	0
Sports Habitus Section	3 (21%)	11 (79%)	0	0	0
Family Sports Culture Section	2 (14%)	10 (71%)	2 (14%)	0	0

Data outside the parentheses indicate the number of experts; data inside represent the corresponding percentage.

Although the expert validity assessment generally endorsed the content measured in this study, it does not necessarily validate that the measurement fully aligns with the research logic. When evaluating specific items, experts often rely on widely accepted conceptual definitions within the field. However, variations in individual familiarity with and depth of understanding of the relevant concepts may introduce subjective judgment biases. Therefore, expert validity should be regarded as a necessary but not sufficient condition for ensuring questionnaire quality. To further examine the validity of the instrument, this study also conducted factor analysis as an additional validation procedure. The items related to physical activity habits in the questionnaire, as well as the family sports culture items from primary school to high school, were numbered in odd and even sequences and divided into two groups. The Spearman correlation coefficient test was conducted on the total scores of each group. The obtained Spearman correlation coefficient was 0.953 (P = 0.000 < 0.01).

To evaluate whether the sample data were suitable for factor analysis, this study employed the Kaiser-Meyer-Olkin (KMO) test to measure sampling adequacy. The principle of this test is to compare the simple correlation coefficients and partial correlation coefficients among variables to determine the extent to which they share a common underlying structure. The KMO statistic ranges between 0 and 1, with higher values indicating a greater proportion of common variance among the variables and thus greater suitability for factor analysis. In this study, a KMO value greater than 0.8 was adopted as the minimum threshold for proceeding with factor analysis.

#### Physical activity habitus: Measurement of adolescent self-reported physical activity capacity and physical behavioral inclination.

Since the factor structure of the scale developed in this study was not yet clearly defined, the research ultimately adopted 8 items to measure adolescents’ physical activity habitus based on existing literature and expert recommendations (see Table 2). Principal component factor analysis was employed to examine the questionnaire’s construct validity. Factor analysis is commonly used to assess scale construct validity, reduce data dimensionality, and establish potential latent variables. The results showed a KMO value of 0.892 (>0.8) and a significant Bartlett’s test of sphericity (p < 0.001), indicating suitability for factor analysis. Two factors were extracted with eigenvalues exceeding the initial communalities (Factor 1 = 4.456; Factor 2 = 1.030). All items demonstrated loadings greater than 0.4 on their corresponding factors, indicating strong variable-factor associations. Based on item content, the two factors were named “self-reported physical activity capacity” and “physical behavioral inclination” respectively. Among these, “You have mastered the basic techniques of a sport and are able to apply these techniques in physical activity” showed the highest loading on the self-reported physical activity capacity dimension, while “You always strive to make time for physical activity” was most representative of the physical behavioral inclination dimension. Physical behavioral inclination reflects an individual’s positive attitude and willingness to participate in physical activities, while physical activity capacity embodies the embodied accumulation of sports skills and knowledge – together these constitute the core dimensions of adolescents’ physical activity habitus.

**Table 2:** Factor Loadings of the measurement indicators of physical activity habitus shaping status by rotating factors.

Physical activity habitus	Measurement indicators	Factor loadings	KMO	Predictive factor scores
Factor1: Physical behavioral inclination	Gx1: Whenever you have time and a place, you will definitely participate in physical activity.	0.731	0.937	0.231
	Gx2: If you don’t engage in physical activity for a week, you feel something is missing in your life.	0.810	0.896	0.392
	Gx3: You always strive to make time for physical activity.	0.854	0.860	0.381
	Gx4: You actively seek suitable sports facilities for participating in physical activity.	0.814	0.895	0.144
Factor2: self-reported physical activity capacity	Gx5: You are able to clearly understand the competition rules of a sport and apply them to guide yourself in physical activity.	0.739	0.910	0.186
	Gx6: You have mastered the basic techniques of a sport and are able to apply these techniques in physical activity.	0.824	0.848	0.276
	Gx7: You can apply common tactics of a sport in physical activity.	0.811	0.865	0.382
	Gx8: You understand basic sports injury prevention and are able to handle any injuries you sustain during physical activity.	0.625	0.945	0.301

KMO = 0.891

#### Family sports culture of primary, middle and high school stages.

This study was based on the main characteristics of family sports culture, and we used principal factor analysis to validate the structural validity of the questionnaire. Study designed six questions and had freshmen and sophomores recall their experiences in primary, middle, and high school to fill out the questionnaire (6 items per school stage, totaling 18 items). Principal factor analysis was then conducted for the six items in each stage. The KMO values for primary school (0.885), middle school (0.907), and high school (0.909) all exceeded the threshold of 0.7, indicating that the data were highly suitable for factor analysis. Furthermore, Bartlett’s Test of Sphericity yielded significant results (p < 0.001) for all education levels, suggesting that the correlation matrix was not an identity matrix and that there were sufficient correlations between the variables to proceed with factor analysis. Therefore, both tests confirmed the adequacy of the data for further analysis. The initial estimation of the common factor variance was derived via the Squared Multiple Correlation (SMC) method. SMC corresponds to the coefficient of determination (R^2^), calculated by regressing each variable against all other variables in the dataset. Elevated SMC values demonstrate a pronounced linear relationship between a specific variable and the remaining variables, thereby indicating enhanced eligibility for inclusion in factor analytic procedures.

As shown in Tables 3–, factor analysis was conducted on the family sports culture measurement items for the primary, middle, and high school stages. Each stage yielded one factor, with eigenvalues (primary factor = 3.786; middle school factor = 4.221; high school factor = 4.181) exceeding the initial communalities (SMC = 0.607, 0.680, 0.767)

**Table 3:** Factor analysis results of the measurement indicators of primary school family sports culture.

Factor	Eigenvalue	Difference	Proportion	Cumulative
Factor1	3.786	3.663	1.038	1.038
Factor2	0.123	0.071	0.034	1.071
Factor3	0.052	0.131	0.014	1.086
Factor4	−0.079	0.022	−0.022	1.064
Factor5	−0.101	0.030	−0.028	1.036
Factor6	−0.131	.	−0.036	1.000

**Table 4:** Factor analysis results of the measurement indicators of middle school family sports culture.

Factor	Eigenvalue	Difference	Proportion	Cumulative
Factor1	4.221	4.142	1.034	1.034
Factor2	0.079	0.059	0.020	1.054
Factor3	0.020	0.075	0.005	1.059
Factor4	−0.055	0.019	−0.014	1.045
Factor5	−0.074	0.038	−0.018	1.027
Factor6	−0.112	.	−0.027	1.000

**Table 5:** Factor analysis results of the measurement indicators of high school family sports culture.

Factor	Eigenvalue	Difference	Proportion	Cumulative
Factor1	4.181	4.114	1.040	1.040
Factor2	0.067	0.055	0.017	1.056
Factor3	0.012	0.059	0.003	1.059
Factor4	−0.047	0.033	−0.012	1.048
Factor5	−0.080	0.032	−0.020	1.028
Factor6	−0.112	.	−0.028	1.000

The factor loadings of the family sports culture measurement items were shown in Table 6. We found that “Family members often participate in sports activities with you.” had a significant impact on the family sports culture measurement indicators for all three stages (primary, middle, and high school). In middle and high school, this indicator had the highest factor loading among all measurement indicators, highlighting the importance of family members participation in physical activities as a core element of family sports culture at this stage. In the primary school stage, although it was slightly less important than “Family often impart you knowledge of physical activity.” it still held a very important position, indicating that cultivating children’s interest in and habitus of participating in sports from an early age forms the foundation of family sports culture. In contrast, the indicator “Family hope you to master physical activity skills” had the lowest factor loading across all three stages. This does not imply that mastering skills is unimportant, but rather reflects that in the construction of family sports culture, family participation and interest cultivation are considered more fundamental and crucial than direct skill expectations. Nevertheless, the score for this indicator was still above the minimum standard, indicating that the learning and development of children’s sports skills remained an important component of family sports culture.

**Table 6:** Factor loadings of the measurement indicators of family sports culture.

Family sports culture	Primary school		Middle school		High school	
	Factor loading	SMC	Factor loading	SMC	Factor loading	SMC
X1: Family members enjoy watching sports events and news	0.708	0.474	0.727	0.628	0.815	0.636
X2: Family members expect you to acquire physical activity skills	0.659	0.409	0.658	0.475	0.699	0.468
X3: More sports equipment at home	0.815	0.644	0.736	0.715	0.848	0.687
X4: Family members often impart you knowledge of sports	0.862	0.707	0.736	0.745	0.881	0.742
X5: Family members often participate in physical activity	0.853	0.710	0.784	0.772	0.889	0.759
X6: Family members often join you in physical activities	0.845	0.703	0.746	0.747	0.860	0.729

### Statistical procedures

This study used family sports culture in the primary, middle, and high school stages as core independent variables. Factor analysis using principal component extraction was applied to obtain the predicted factor scores for family sports culture at each stage, which were then used as the variable values for the family sports culture variables at each stage. The family sports culture variables for the primary, middle, and high school stages were all continuous variables.All statistical analyses were performed using Stata 16.0. We conducted exploratory factor analysis (EFA) to validate the scale’s construct validity and employed ordinary least squares (OLS) regression models to test the research hypotheses.

Given that the core dependent variable of this study (family sports culture) is an approximately continuous variable, the study employed the ordinary least squares regression model to verify the possible causal relationship between family sports culture and teenagers’ sports activity habits. Moreover, gender, urban-rural classification, family economic status across various educational stages, parental education levels, and parental occupations were collectively included as control variables in the model for analysis. The specific model settings were as follows:


$$y_{i}\;=\;\alpha_{\text{0}}\;+\;\alpha_{\text{1}}x_{\text{1}}\;+\;\alpha_{\text{2}}x_{\text{2}}\;+\;\alpha_{\text{3}}x_{\text{3}}\;+\;\beta_{\text{1}}D_{\text{1}}\;+\;\beta_{\text{2}}D_{\text{2}}\;+\;\beta_{\text{3}}D_{\text{3}}\;+\;\beta_{\text{4}}D_{\text{4}}\;+\;\beta_{\text{5}}D_{\text{5}}\;+\;\beta_{\text{6}}D_{\text{6}}\;+\;\beta_{\text{7}}D_{\text{7}}\;+\;\epsilon$$

**Fig 1:**
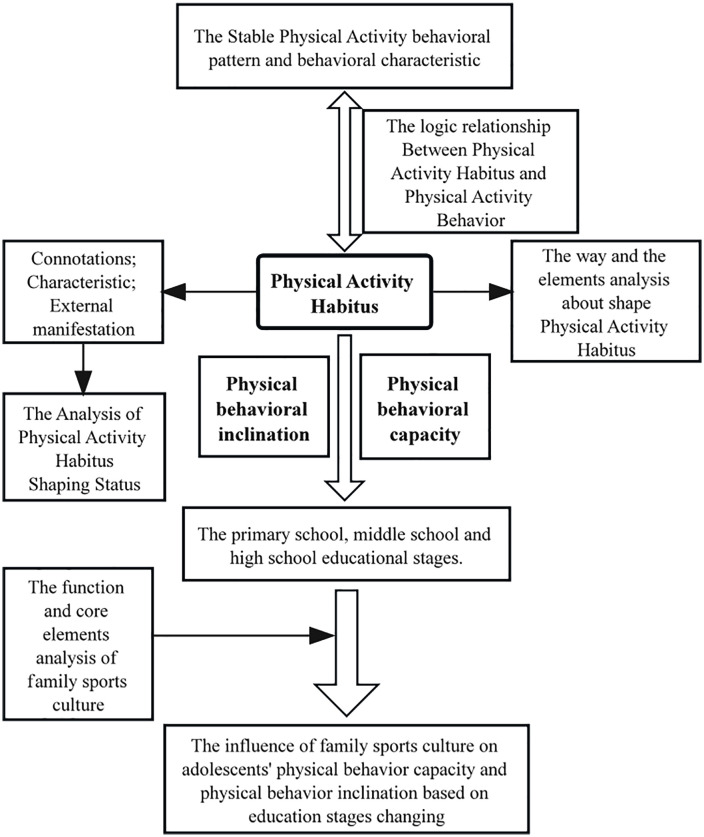
The research organization.

*y*_*i*_(*i* = 1,2) represented self-reported physical activity capacity and physical behavioral inclination as the dependent variables. *x*_*i*_(*i* = 1,2,3) *y*_*i*_(*i* = 1,2) was the kernel independent variable, respectively represents the family sports culture of primary school, middle school and high school period. *D*_*i*_(*i* = 1,2,...7) was a dummy variable that represents gender, difference between urban and rural, parental education level, family economic status across primary school, middle school and high school. The research framework was illustrated in Fig 1.

## Results

### The shaping effect of family sports culture at each educational stage on self-reported physical activity capacity

To examine the influence of family sports culture across different educational stages on adolescents’ self-reported physical activity capacity, the study established four regression models. First, evaluation of the final model (Model 1) demonstrated acceptable overall model fit, with the complete model explaining 21.9% of the variance in the outcome variable (R^2^ = 0.219), with detailed results presented in Table 7. Furthermore, the model passed both residual normality and homoscedasticity tests.

**Table 7:** OLS regression analysis of the shaping effect of family sports culture at each educational stage on self-reported physical activity capacity.

Self-reported physical activity capacity	Model 1 (R^2^ = 0.219)			Model 2 (R^2^ = 0.209)			Model 3 (R^2^ = 0.201)			Model 4 (R^2^ = 0.191)		
	β	95% confidence intervals		β	95% confidence intervals		β	95% confidence intervals		β	95% confidence intervals	
Family sports culture at primary school stage	0.226^***^	0.167	0.284	0.375^***^	0.342	0.407						
Family sports culture at middle school stage	0.089^**^	0.009	0.170				0.357^***^	0.325	0.388			
Family sports culture at high school stage	0.088^***^	0.020	0.158							0.339^***^	0.307	0.370
Gender	0.367^***^	0.310	0.426	0.371^***^	0.313	0.430	0.373^***^	0.314	0.432	0.372^***^	0.313	0.431
Rural & Urban	0.071^**^	0.004	0.138	0.077^**^	0.010	0.145	0.071^**^	0.003	0.139	0.077^**^	0.009	0.146
Family economic status at primary school stage	0.027	−0.040	0.096	0.057^**^	0.010	0.105						
Family economic status at middle school stage	0.012	−0.092	0.115				0.082^***^	0.027	0.136			
Family economic status at high school stage	0.042	−0.041	0.125							0.079^***^	0.025	0.133
Father’s education level	−0.034^*^	−0.072	0.003	−0.030	−0.040	0.035	−0.027	−0.065	0.011	−0.018	−0.057	0.019
Mother’s education level	−0.005	−0.042	0.033	−0.002	−0.898	−0.546	−0.003	−0.034	0.041	−0.006	−0.020	0.055
Mean VIF	3.26			1.41			1.40			1.39		

***means statistical significance at the 1% level, ** means statistical significance at the 5% level, * means statistical significance at the 10% level; VIF stands for Variance Inflation Factor, and the Mean VIF serves as an indicator for assessing the severity of multicollinearity in regression models containing multiple independent variables. Generally, a Mean VIF value below 5 is considered an acceptable threshold for the model.

The results (see Table 7) indicated that family sports culture served as a stable positive predictor of adolescents’ self-reported physical activity capacity, though its effects demonstrated notable stage-specific heterogeneity. Specifically, its influence was strongest during primary school (β = 0.226, p < 0.01), with a significantly larger effect size compared to middle and high school stages. This finding highlights the crucial role of early family intervention. Among control variables: male gender (β range: 0.367–0.373) and urban household registration (βrange: 0.071–0.077) consistently showed moderate positive predictive effects. A noteworthy finding emerged regarding paternal education level, which demonstrated a slight negative effect (β = −0.034, p < 0.1). Model comparisons further revealed that when family sports culture was incorporated into the full model (Model 1), the independent effect of family economic status was attenuated, while it remained significant in stage-specific models. This pattern suggests that family sports culture may function as an independent driver of adolescent sports competence, operating through pathways distinct from economic conditions.

### The shaping effect of family sports culture at each educational stage on physical behavioral inclination

To examine the influence of family sports culture across different educational stages on adolescents’ physical behavioral inclination, this study established four regression models. The evaluation of the full model (Model 1) demonstrated an acceptable overall model fit. The complete model explained 14.2% of the variance in the outcome variable (R^2^ = 0.142) and passed both residual normality and homoscedasticity tests, with detailed results presented in Table 8.

**Table 8:** OLS regression analysis of the shaping effect of family sports culture at each educational stage on physical behavioral inclination.

Physical behavioral inclination	Model 1 (R^2^ = 0.142)			Model 2 (R^2^ = 0.133)			Model 3 (R^2^ = 0.125)			Model 4 (R^2^ = 0.123)		
	β	95% confidence intervals		β	95% confidence intervals		β	95% confidence intervals		β	95% confidence intervals	
Family sports culture at primary school stage	0.211^***^	0.149	0.273	0.337^***^	0.303	0.372						
Family sports culture at middle school stage	0.027	−0.059	0.113				0.318^***^	0.284	0.351			
Family sports culture at high school stage	0.130^***^	0.056	0.203							0.310^***^	0.276	0.343
Gender	0.258^***^	0.196	0.320	0.262^***^	0.200	0.325	0.265^***^	0.203	0.328	0.268^***^	0.206	0.331
Rural & Urban	−0.114^***^	−0.187	−0.042	−0.110^***^	−0.183	−0.038	−0.115^***^	−0.187	−0.042	−0.113^***^	−0.186	−0.041
Family economic status at primary school stage	−0.006	−0.078	0.067	−0.038	−0.089	0.013						
Family economic status at middle school stage	−0.092	−0.203	0.018				−0.044	−0.102	0.014			
Family economic status at high school stage	0.049	−0.039	0.138							−0.013	−0.070	0.044
Father’s education level	−0.044^**^	−0.085	−0.003	−0.044^**^	−0.084	−0.003	−0.038^*^	−0.079	0.003	−0.035^*^	−0.076	0.006
Mother’s education level	−0.033	−0.073	0.007	−0.035	−0.075	0.008	−0.027	−0.068	0.013	−0.019	−0.059	0.021
VIF Mean	3.26			1.41			1.40			1.39		

***means statistical significance at the 1% level, ** means statistical significance at the 5% level, * means statistical significance at the 10% level; VIF stands for Variance Inflation Factor, and the Mean VIF serves as an indicator for assessing the severity of multicollinearity in regression models containing multiple independent variables. Generally, a Mean VIF value below 5 is considered an acceptable threshold for the model.

The results demonstrated that family sports culture served as a stable positive predictor of adolescents’ physical behavioral inclination, though its effects exhibit significant stage-specific heterogeneity. Specifically, family sports culture showed the strongest positive predictive effect during primary school (β = 0.211, p < 0.01), while its influence in middle school was not statistically significant in Model 1. Regarding control variables, male adolescents (β range: 0.258–0.268) and students with rural household registration (β range: −0.110 to −0.115) generally demonstrated higher physical behavioral inclination. Consistent with the self-reported physical activity capacity model, paternal education level again showed a stable negative effect (β = −0.048, p < 0.05), suggesting that within the population surveyed in this study, highly educated fathers may not have played an active role in encouraging their children’s physical activity participation.

It is worth noting that while family sports culture demonstrated an independent positive effect on adolescents’ physical behavioral inclination during middle school in the separate model (Model 3), this influence was overshadowed by the stronger primary school effect in the full model (Model 1). This pattern suggests that early-established family sports culture may create a stable behavioral foundation, thereby diminishing the marginal utility of subsequent cultural investments. Consequently, the impact of family sports culture does not follow a simple cumulative mechanism but rather represents a process dominated by early formation with sustained effects, highlighting the critical window of opportunity for cultivating family sports atmosphere during primary school.

## Discussion

This study employed a retrospective survey methodology, investigating potential causal relationships between family sports culture and adolescents’ physical activity habitus through university freshmen and sophomores’ recollections of their experiences from primary to high school education stages. Factor analysis empirically demonstrated that adolescent sports habitus can be effectively measured through two dimensions: self-reported physical activity capacity and physical behavioral inclination. Regression analysis further revealed that family sports culture may positively influence both self-reported physical activity capacity and physical behavioral inclination across different educational stages, with effect strengths showing distinct variations: most pronounced during primary school, diminished in middle school, and intermediate in high school. Notably, this study confirms that actively cultivating family sports culture helps mitigate disparities in adolescents’ self-reported physical activity capacity and physical behavioral inclination resulting from unfavorable family economic conditions, highlighting the crucial role of family sports culture development in promoting health equity and adolescents’ behavioral formation. Additionally, higher paternal education levels demonstrated negative correlations with adolescent sports habitus development, potentially reflecting the tendency among highly educated families in certain Chinese regions to concentrate resources on academic competition.

This empirical study reaffirms Bourdieu’s perspective that behavioral capacity must be fully considered when measuring habitus. Habitus is rooted in family contexts [38]. Research on physical activity habitus must focus on the role of family. Numerous researchers have suggested that parental education level, economic status, and social position influence adolescents’ participation in physical activities. The general trend indicates that adolescents from families with better economic conditions and higher social status participate in physical activities at higher rates [39]. A British study indicated that young athletes’ participation in high-level sports largely depends on their parents, while some talented youths face significant disadvantages due to unfavorable family economic circumstances [40]. Although students from higher social status backgrounds participate in physical activities more frequently and are more likely to join sports clubs, it is habits, parental sports environments, and sports partners that serve as the primary factors driving their regular participation in physical activities [41]. Therefore, families with lower socioeconomic status should place greater emphasis on family sports culture. It is worth noting that the findings of this study reveal that family economic status is not the decisive factor determining adolescents’ physical activity behaviors. A positive family sports culture can, to some extent, mitigate the adverse effects of poor family economic conditions and similarly predict higher levels of physical activity habitus. However, since this study focuses on general students, whether effective family sports culture remains equally impactful on athletes’ physical activity habitus requires further discussion.

Furthermore, this study identified that family sports culture demonstrates the most pronounced predictive effect on physical activity habits during primary school—a phenomenon well-aligned with developmental principles and habitus formation mechanisms. The primary school stages coincide with a critical window for establishing behavioral patterns, when children experience lighter academic pressure, earlier school dismissal, and spend more time in family environments. During this phase, children’s stronger dependency on family and underdeveloped independent thinking make them particularly receptive to internalizing family sports practices as enduring embodied dispositions through daily interactions. As adolescents transition into puberty, their social focus shifts toward peer groups and school environments. The development of self-esteem, autonomy, and independent judgment—coupled with subconscious resistance to parental influence during adolescent rebellion—collectively diminishes the marginal utility of family cultural inputs. Meanwhile, the habitus formed during primary school continues to exert persistent effects, jointly contributing to diminishing returns on family sports investments during middle school. These findings collectively suggest that primary school represents the crucial intervention window for leveraging family sports culture to promote adolescent physical development.

Some existing studies suggest that the frequency of participation in sports and physical activities may gradually decrease from childhood through adolescence to adulthood [42]. Moreover, as adolescents grow older, the popularity of physical education courses tends to decline [43]. Furthermore, with increasing academic pressure, highly educated parents’ own test-oriented educational experiences during their youth may lead them to prioritize supplementary tutoring over extracurricular physical activity for their children. This also explains the negative correlation observed in this study between higher paternal education levels and adolescent sports habits. These findings further validate that secondary school students generally face greater barriers to physical activity than primary students, with educational stage serving as a determining factor in sports disengagement [44].

Current academic exploration of habitus theory remains predominantly qualitative. This study draws upon previous research in the field of habitus theory while employing quantitative methods to measure adolescents’ physical activity habitus. Our research enriches habitus theory and addresses the empirical research gap concerning physical activity habitus, thereby providing a practical framework for understanding this abstract concept. From a practical perspective, it not only helps the public develop scientific physical activity lifestyles through understanding physical activity habitus, but also offers concrete theoretical references for future scholars seeking to operationalize and measure physical activity habitus.

### Limitations and further direction

As argued above, our results were likely to have a causal interpretation due to the empirical strategy employed. Some data limitations, however, indicated a need for further investigation in future research. This study aimed to measure family sports culture from primary school to high school. Given the difficulty of conducting a longitudinal study over such an extended period, the method used in this study involved having first- and second-year university students recall their past experiences to complete the survey, which was a form of retrospective study. Although this study has been carefully designed and optimized based on expert recommendations and pre-survey results, we are still exploring ways to improve. In future research, we plan to adopt a longitudinal tracking approach to further enhance the accuracy of the data. Due to the limited scope of the research object, the obtained data were solely derived from college students enrolled in 16 universities located in Chongqing, China. The empirical research was restricted to Chongqing, which may result in some deviations when compared with college students from other regions. It was necessary to include more samples in the following research to enhance the reliability of the research. Moreover, it is worth noting noted that when applying the suggestions discussed in this paper regarding adolescent physical habitus to students in other countries or regions, adjustments must be made according to their specific social contexts. Furthermore, our future endeavors will encompass an expanded scope of research subjects including students from Chinese southwest areas, eastern coastal areas, western regions, or even nationwide for in-depth investigations into how to shape physical habitus. It is important to acknowledge that physical habitus is socially and historically determined. However, the existing research only focused on the impact of early family sports culture on the self-reported physical activity capacity and inclination at its current stages, while neglecting the effect on shaping subsequent physical habitus. Thus, it remained unexplored whether family sports culture during basic education periods significantly impacts an individual’s lifelong physical habitus. Further research and discussion are necessary to reach a mutually agreed conclusion about this matter.

When it comes to defining physical habitus, this study primarily focused on the theoretical deduction of self-reported physical activity capacity and physical behavioral inclination. Translating the abstract theoretical construct of “sports habitus” into measurable empirical indicators presents a key methodological challenge. Our measurement strategy does not aim to capture the full conceptual depth of “sports habitus” directly. Instead, it adheres to standard operationalization procedures in social science research by focusing on its observable manifestations and outcomes—specifically, individuals’ behavioral tendencies and physical competencies within specific social fields—thereby indirectly yet effectively approximating the latent concept. Further researches are required to ascertain whether these two dimensions alone suffice for a comprehensive explanation of physical habitus. However, through the validation of this study, we have substantiated that the family sports culture, as measured by our proposed framework, has a cause-and-effect relationship with the cultivation of physical habitus. Therefore, this measurement scheme held meaningful theoretical and practical implications. It is also recommended that future research endeavors delve deeper into the measurement indices of physical habitus, and subsequently embark on a more scientific and systematic exploration of the intricate relationship between physical habitus and family sports culture. Moreover, corresponding intervention strategies should be formulated to promote the healthy growth and all-round development of adolescents.
